# Mechanisms and FEM Simulation of Chip Formation in Orthogonal Cutting In-Situ TiB_2_/7050Al MMC

**DOI:** 10.3390/ma11040606

**Published:** 2018-04-15

**Authors:** Yifeng Xiong, Wenhu Wang, Ruisong Jiang, Kunyang Lin, Mingwei Shao

**Affiliations:** The Key Laboratory of Contemporary Design and Integrated Manufacturing Technology, Ministry of Education, Northwestern Polytechnical University, Xi’an 710072, China; xiongyifeng@mail.nwpu.edu.cn (Y.X.); npuwwh@nwpu.edu.cn (W.W.); linkunyang@mail.nwpu.edu.cn (K.L.); 2017261152@mail.nwpu.edu.cn (M.S.)

**Keywords:** in-situ, TiB_2_ particle, Al metal matrix composite, chip formation, FEM simulation

## Abstract

The in-situ TiB_2_/7050Al composite is a new kind of Al-based metal matrix composite (MMC) with super properties, such as low density, improved strength, and wear resistance. This paper, for a deep insight into its cutting performance, involves a study of the chip formation process and finite element simulation during orthogonal cutting in-situ TiB_2_/7050Al MMC. With chips, material properties, cutting forces, and tool geometry parameters, the Johnson–Cook (J–C) constitutive equation of in-situ TiB_2_/7050Al composite was established. Then, the cutting simulation model was established by applying the Abaqus–Explicit method, and the serrated chip, shear plane, strain rate, and temperature were analyzed. The experimental and simulation results showed that the obtained material’s constitutive equation was of high reliability, and the saw-tooth chips occurred commonly under either low or high cutting speed and small or large feed rate. From result analysis, it was found that the mechanisms of chip formation included plastic deformation, adiabatic shear, shearing slip, and crack extension. In addition, it was found that the existence of small, hard particles reduced the ductility of the MMC and resulted in segmental chips.

## 1. Introduction

Many attempts have been made in the past few decades to obtain a deep understanding of and create a model of the metal cutting process by using analytical, experimental, mechanistic, and finite element methods [1]. Remarkable work has been made by Merchant [2] to determine the shear angle using an analytical model. Lee and Shaffer [3] and Oxley and Hatton [4] proposed analytical models for predicting the shear angle based on an assumption of a thick shear zone.

On the basis of shear plane theory, Das and Tobias [5] developed a mathematical model describing the connection between static and dynamic cutting force coefficients with experimental methods. Wu [6] proposed a new approach for obtaining dynamic cutting process parameters, using the time series based on dynamic data.

For an insight into the physical origins and dynamic phenomena, Lopez de Lacalle et al. [7] developed a data acquisition system to simultaneously record the tool position and cutting forces for correlating machined surface geometry and cutting forces. By applying the mechanistic method, Armarego and Deshpande [8], Kolartis and DeVries [9], and Lazoglu and Liang [10] established dynamic models of peripheral end milling and ball-end milling, respectively. In their models, the effect of system deflections on the chip load was taken into consideration.

Based on Armerego’s idea, Fernandez-Abia et al. [11] took into consideration tool nose radius and proposed expressions for determining shearing and edge cutting coefficients for a wide range of cutting conditions. Lamikiz et al. [12] presented a new method of obtaining the shear and ploughing specific cutting coefficients in ball-end milling. Wan et al. [13] presented a unified, instantaneous cutting force model for flat end mills, using a transformation approach from orthogonal to oblique cutting process from Altintas [14] and Armarego and Whitfield [15].

The finite element method (FEM) is an effective approach in the study of cutting process modeling [16]. Scippa et al. [17] proposed a novel finite element approach (FEA) for the thin-wall milling process considering the effects of fixturing, tooltip dynamics, and material removal. This also showed that the finite element method could simulate the milling process. With the finite element method, Gonzalo et al. [18,19] proposed a new and inexpensive approach for obtaining cutting force coefficients using FEM models instead of cutting experiments.

The FEM has also been applied in the process modeling of Al-metal matrix composites (Al-MMCs), which have been one of the most important metal matrix composites, with low density, low cost, and increased strength and wear resistance [20,21]. To date, as a typical representative, the ex-situ SiC_p_/Al MMCs have been widely and deeply researched [22,23] due to their relatively simple preparation process and low demand for equipment. During the cutting process, the chip morphology and segmentation was found to have a dominant influence on the material machinability [24]. Hence, importance and significance has been attached to the analysis of chip formation in cutting process modeling for studying the mechanism of material machining deeply. Much research has been reported on the chip formation of ex-situ SiC_p_/Al MMCs with experimental and finite element methods [25,26].

With turning tests on A359/SiC/20_p_ composite, Lin et al. [27] found that tool wear had a great influence on the nature of the formed chip. In addition, it was concluded that the short chips were formed due to the reduction of material ductility, which resulted from the presence of ceramic particles.

Ge et al. [28] found that chips of SiC_p_/Al composite were formed non-uniformly and that the chip formation mechanisms, which were different from the adiabatic shear of titanic alloy, involved the micro-voids, micro-cracks, and strain concentration in the shear plane. Similarly, it was noted by Du et al. [29] that the debonding failure of the particle-matrix interface, cracks and voids all played a vital role in the chip formation process.

Karthikeyan et al. [30] studied the impact of particle volume fraction and cutting parameters on chip formation. It was found that the chip transformed from a continuous type to a discontinuous type with increasing particle volume fraction. With a decrease in feed or an increase in speed, continuous chip formation seemed to be easier due to the increased material ductility.

Huang et al. [31] divided chips into three categories: Al-matrix, SiC particle, and Al–SiC mixed chips in grinding high-volume SiC_p_/Al composites. It was found that the Al-matrix and SiC particle chips formed similarly due to plastic deformation and material brittleness. Reddy et al. [32] also noted that hard particles in the matrix might decrease material ductility, which resulted in discontinuous, serrated, and saw-toothed chips. Joshi et al. [33] found that the fracture initiated at the outer chip surface and propagated to the tool nose.

However, with only experimental tests and empirical methods as discussed above, it is difficult to penetrate the complex deformation process, such as elastic–plastic deformation, strain and stress, and temperature in the deformation zone and interactions, which could provide comprehensive and strong understanding. Hence, a large amount of deep research has also been carried out into understanding chip formation mechanisms during the cutting of SiC_p_/Al composites by applying sophisticated numerical techniques like the FEM.

Applying a 2D finite element (FE) model, Guo et al. [34] conducted a study on the effect of cutting speed and tool rake angle on chip morphology in machining SiC_p_/Al MMC. In their model, the material property was described with the Johnson–Cook (J–C) model. In addition, the arbitrary Lagrangian–Eulerian (ALE) method and the J–C damage model were both applied to define the chip separation criterion. The results showed that the rake angle and speed had a great impact on chip morphology. However, the formation process of the saw-toothed chip, which was commonly observed in the above experimental studies, was not presented.

Fathipour et al. [35] built a 2D FE model and found that the chip separation usually performed along the particle–matrix interfaces. In addition, the chips were commonly saw-toothed, and the cutting parameters had a significant influence on the size and distance of the saw-tooth feature.

Wang et al. [36] built multi-phase models of SiC_p_/Al composites of different particle volume fractions. They believed that the debonding of the particle–matrix interfaces would take away part of the plastic deformation force, which would result in the decrease of cutting forces. From their simulation results, it was also found that chips of SiC_p_/Al composites were segmented and would become more discontinuous as the particle volume fraction increased. Shui et al. [37] developed three models with different particle types for simulating the cutting process of SiC_p_/2024Al composites and found that chips of SiC_p_/2024Al were mainly pieces or powder.

Up to now, a large number of papers have been published on ex-situ SiC_p_/Al MMCs. However, research on topics such as cutting performance, tool wear, machining modeling, or simulation on in-situ TiB_2_/7050Al MMC is rarely reported. In this study, the orthogonal cutting experiments and the FEM simulation methods were applied to investigate the chip formation mechanism of a new kind of in-situ TiB_2_/7050Al metal matrix composite. With experimental data, the influence of cutting parameters on chip geometric parameters was analyzed. In addition, the J–C constitutive equation was identified from the basic orthogonal cutting tests and validated with experiments and simulation tests. The stress, strain, temperature, and chip formation mechanisms around the material deformation zone were studied with the proposed FE simulation model.

## 2. Materials and Methods

### 2.1. Tool and Workpiece

**Figure 1:**
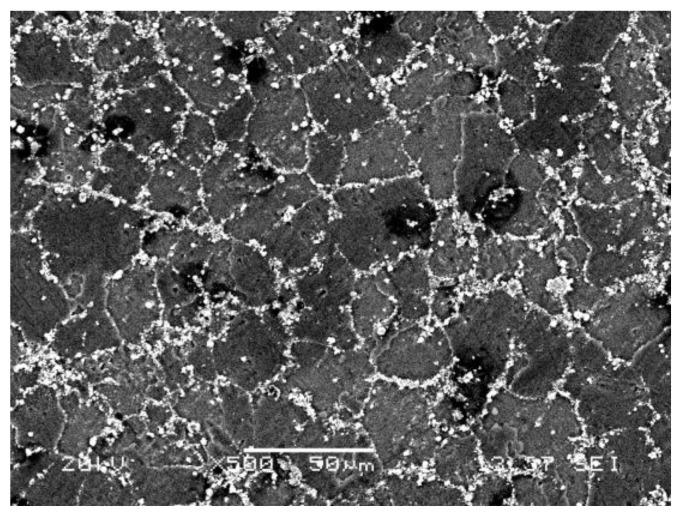
Microstructure of TiB_2_/7050Al MMC.

In the previous studies [38,39,40], the polycrystalline diamond (PCD) tool was proved to be the ideal tool for cutting MMCs. In this study, the specifications of the PCD tool used is presented in Table 1. Table 2 presents the material properties of the PCD tool. The workpiece used was in-situ 6 wt % TiB_2_/7050Al MMC, for which the chemical composition of the matrix material and the material physical properties are shown in Table 3 and Table 4, respectively. With the in-situ synthesis method, the particle–matrix interfaces are clean, and the TiB_2_ particles, which range in size from 50~200 nm as shown in Figure 1, are evenly distributed.

### 2.2. Experiment and Measurement

**Figure 2:**
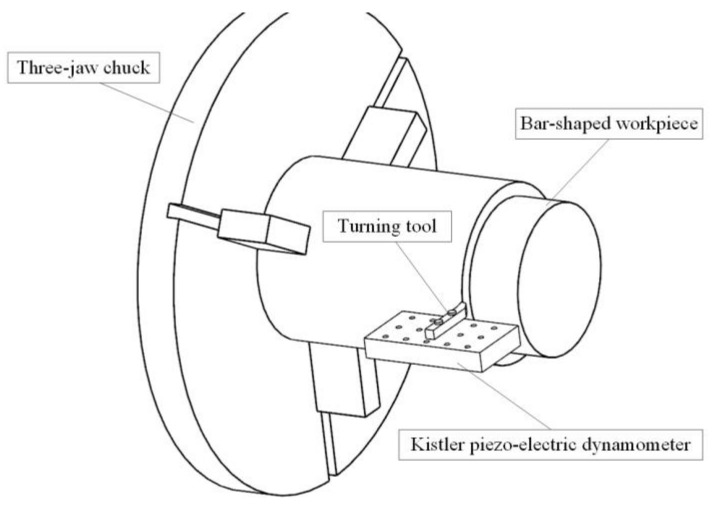
Orthogonal cutting experiments on the CK7525 CNC Lathe.

The orthogonal cutting experiments were carried out on a CK7525 CNC Lathe (Bochi Machine Tool Group Co. Ltd., Baoji, China) under dry condition as presented in Figure 2. Bar-shaped workpieces of 140 mm in diameter and 200 mm in length were used, and the cutting forces were measured with a Kistler 9255B dynamometer (Kistler ,Winterthur, Wwitzerland).

An orthogonal cutting test, which included three factors and five levels, was performed to determine the material’s constitutive equation. A set of single-factor tests was also carried out to study the influence of the cutting parameters on chip formation and to validate the simulation model. The detailed cutting parameters are listed in Table 5. For each test, the cutting process lasted for over 15 seconds. After cutting, the chips were collected, and the chip thickness was measured three times with a micrometer. A scanning electron microscope (SEM) (Tescan China, Shanghai, China) and three-dimensional surface profilometer were used for investigating and measuring the chip morphology and geometry parameters.

**Figure 3:**
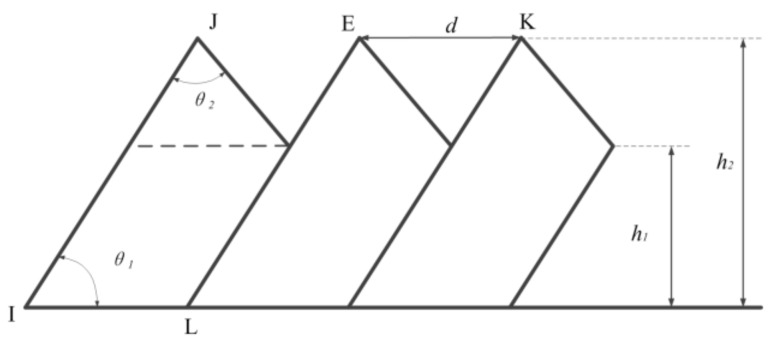
Geometric parameters of the saw-tooth chip.

As presented in Figure 3, the deformation of a saw-tooth chip is uneven. Generally, there are several parameters used to describe the geometrical characteristics of saw-tooth chips, such as tooth top and root height (*h*_2_, *h*_1_), tooth vertex angle and basic angle (*θ*_2_, *θ*_1_), and pitch *d*.

The saw-tooth degree *G_s_*, the frequency of saw-tooth *f*, and the pitch *d* are usually used to represent and analyze the chip’s geometrical characteristics, which could be obtained using following equations:

(1) $$G_{s}=\frac{h_{2}-h_{1}}{h_{2}}$$

(2) $$f=\frac{v_{s}\sin\emptyset }{d\sin\theta_{1}}$$

where *φ* stands for the shear angle that could be obtained with Merchant’s theory [2].

## 3. Numerical Modeling

In this study, a two-dimensional FE model was built for chip formation simulation. As there is no constitutive equation for this new kind of in-situ 6 wt % TiB_2_/7050Al MMC, the J–C constitutive equation was applied to model its thermo-elastic-plastic behavior at present. Therefore, a set of orthogonal cutting experiments was conducted to obtain some material parameters needed for the J–C constitutive equation.

### 3.1. Definition of Material Constitutive Equation

The J–C constitutive equation used for modeling the thermo-elastic-plastic behavior of the in-situ TiB_2_/7050Al MMC could be described as below:

(3) $$\sigma =\left[A+B{\left(\varepsilon \right)}^{n}\right]\left[1+C\ln\left(\frac{\overset{´}{\varepsilon }}{\overset{´}{\varepsilon_{0}}}\right)\right]\left[1-{\left(\frac{T-T_{0}}{T_{melt}-T_{0}}\right)}^{m}\right]$$

where *σ* is the flow stress and *ε*, $\overset{´}{\varepsilon }$, $\overset{´}{\varepsilon_{0}}$ means the effective plastic strain, the effective strain rate, and the reference strain rate (10^−3^/s in this study), respectively. *T*_0_ and *T_melt_* stands for the room temperature and material melting temperature. The coefficients *A*, *B*, *C*, *m*, and *n* stand for the yield strength, hardening modulus, strain rate sensitivity coefficient, thermal softening coefficient, and hardening coefficient, respectively.

In order to obtain the constants (*A*, *B*, *C*, *m*, and *n*), the physical quantities on the shear plane (*σ*, *ε*, $\overset{´}{\varepsilon }$, and *T*) should be determined. From the research of Ref. [41], the flow stress *σ*, the effective plastic strain *ε*, and the effective strain rate $\overset{´}{\varepsilon }$ could be determined on the basis of orthogonal cutting test results by the following formulas:

(4) $$\sigma =\sqrt{3}\left|\tau_{shear}\right|$$

(5) $$\overset{´}{\varepsilon }=\frac{2v_{s}\cos\gamma }{\sqrt{3}h\cos\left(\emptyset -\gamma \right)}$$

(6) $$\varepsilon =\frac{\cos\gamma }{\sqrt{3}\cos\left(\emptyset -\gamma \right)\sin\emptyset }\left(\frac{1}{2}+\frac{\cos\left(2\emptyset -\gamma \right)}{2\cos\gamma }\right)$$

(7) $$T=T_{0}-\left[\left(\frac{1}{2}+\frac{\cos\left(2\emptyset -\gamma \right)}{2\cos\gamma }\right)\frac{\cos\gamma }{\rho C_{p}\cos\left(\emptyset -\gamma \right)\sin\emptyset }\right]\left[\frac{2\tau_{shear}+\tau_{0}}{3}\right]$$

where *γ*, *h*, and *τ_shear_* stands for tool rake angle, primary shear zone thickness, and shear stress, respectively, which could be obtained from Merchant’s theory. In addition, *ρ* is the mass density and *C_p_* is the specific heat.

With determined *σ*, *ε*, $\overset{´}{\varepsilon }$, and *T*, the five constants could be obtained using the least-square approximation (LSA) method. However, it was noticed in some studies [42] that the least-square approximation method showed poor convergence and greatly depended on the searching point and boundary constraints. Hence, the genetic algorithm (GA) was applied to determine the constants by globally optimizing the following equation:

(8) $$f\left(A,B,C,m,n\right)=min\left\{{\left||\frac{A+B\varepsilon^{n}}{\sqrt{3}\left|\tau_{shear}\right|}\left[1+C\ln\left(\frac{\overset{´}{\varepsilon }}{\overset{´}{\varepsilon_{0}}}\right)\right]\left[1-{\left(\frac{T-T_{0}}{T_{melt}-T_{0}}\right)}^{m}\right]-1|\right|}_{\infty }\right\}$$

**Figure 4:**
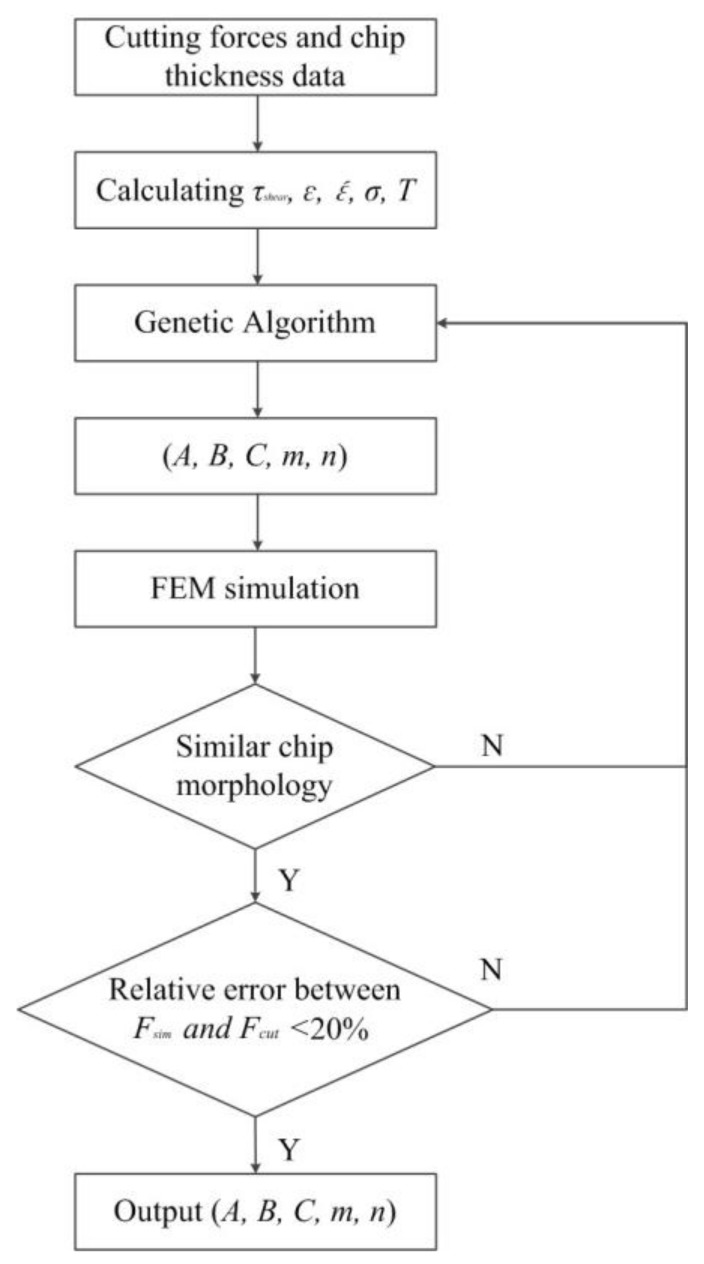
Flowchart of defining the material constitutive equation.

The material constitutive equation could be obtained after the aforementioned computation that was also presented with a flowchart in Figure 4. As presented, the material constitutive constants (*A*, *B*, *C*, *m*, and *n*) were output only when the chip morphology was similar and the relative error of the cutting force was less than 20%. After that, the obtained material constitutive parameters presented in Table 6 were input into the simulation model. Then, the chip morphology and cutting forces of the FEM simulation and orthogonal cutting experiments were analyzed under the same cutting conditions for model validation.

### 3.2. Two-Dimensional Modeling

**Figure 5:**
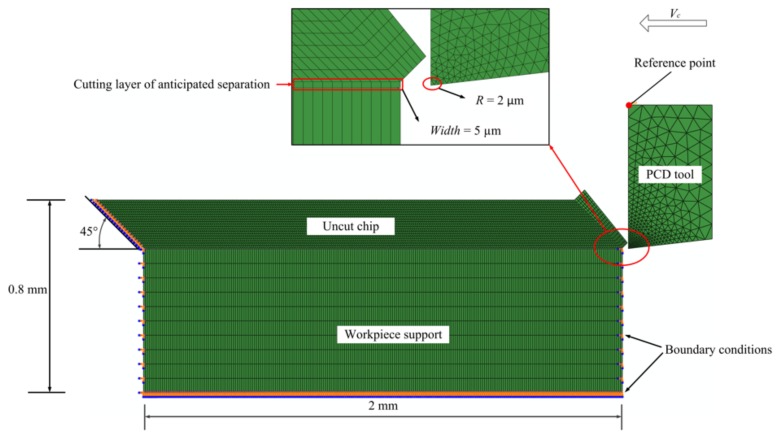
Geometric model for two-dimensional (2D) orthogonal cutting process simulation.

The commercial finite element simulation software Abaqus was applied in our research. In order to avoid the contact convergence and improve the physical comprehension of the chip formation, the Abaqus–Explicit approach was chosen for simulation analysis. As shown in Figure 5, a two-dimensional coupled temperature-displacement analysis model was developed for the orthogonal cutting process simulation.

Because the hard PCD tool was used, whose elastic modulus was much larger than that of the workpiece, the tool was defined as an analytical rigid. The gradient grid method was used in meshing the cutting tool part, and the element density of the tool nose was much larger than that of other parts, as shown in Figure 5. The total number of elements and nodes of the cutting tool was 599 and 338, respectively.

The workpiece model consisted of three parts: (a) the predefined uncut chip; (b) the cutting layer; and (c) the workpiece support. Due to the small volume (6 wt %) and nanometer size (50–200 nm), it was very difficult to create an uncut chip part with 6 wt % small particles and to define the interaction between the particles and the matrix material even by python programming. In addition, due to the huge size difference between the particle size (nanometer) and the uncut chip thickness (millimeter), the submitted simulation job was aborted easily due to mesh distortion, interaction relationship, and tremendous computing workload. Therefore, in our study, the uncut chip part was created as an isotropic monophasic part without particles.

The uncut chip thickness was set as 0.2 mm. In order to make sure the chip elements could climb up along the rake face successfully, the uncut chip part was modeled to a parallelogram with a trapezoidal head, as shown in Figure 5. Linear quadrilateral continuum plane strain element with reduced integration (CPE4RT), distortion control, and hourglass control were used in meshing the predefined uncut chip part, and the mesh element was also a parallelogram with a length-to-height ratio of 8:10. The number of elements and nodes of the predefined uncut chip were 5120 and 5397, respectively.

In our simulation model, a cutting layer along the path of anticipated separation was used as the sacrificial layer. To avoid mesh distortion, the width of the cutting layer element should be larger than the cutting edge radius, and it was set as 5 μm in this study. For boundary conditions, the workpiece part was fixed on its bottom, left, and right sides, as well as the left side of the uncut chip part. The rigid tool part was designed to move from the right to the left at a range of cutting speeds (50 m/min–450 m/min) along the cutting layer.

### 3.3. Chip Separation Criterion and Chip–Tool Interface

It is very important to define the material failure criteria in the simulation of the material cutting process. According to the max stress, strain, and energy theory, there are many material failure criteria that could be used in the cutting process simulation, such as the J–C failure criterion, the shear damage, the ductile damage, and so on.

The J–C failure criterion is widely used in isotropic metal cutting simulations. Unfortunately, we could not obtain the J–C failure criterion for this new kind material from simple cutting tests. Even though it is possible to obtain the J–C failure criterion, it would cost months to perform a set of damage experiments with an expensive device. Hence, based on the shear damage, a chip separation criterion was developed using the shear failure module in our study:

(9) $$D=\frac{\varepsilon_{pl}}{\varepsilon_{pl}^{d}}=1$$

where *ε_pl_* means the equivalent plastic strain, $\varepsilon_{pl}^{d}$ means the damage plastic strain, and *D* stands for the damage parameter. Only if the damage parameter equals 1 would the material fail and the mesh be deleted.

There are two contact zones in the simulation model between the cutting tool and the workpiece: (a) the contact between the rake face and the chip; (b) the contact between the rake face and the machined surface. Friction played an important role in the material cutting. The Coulomb friction law was applied to define the contact relationship as follow:

(10) $$\tau_{c}=\min\left(\mu p,\tau_{th}\right)$$

where *τ_c_* and *τ_th_* stand for the critical friction stress and the threshold value of the material failure, respectively. The parameter *p* is the normal pressure across the contact interface, and *μ* is the friction coefficient. As the PCD tool was very sharp and little serious adhesive wear phenomenon was found during the cutting experiments, the friction coefficient *μ* was set as 0.1 in our simulation.

## 4. Results and Discussion

### 4.1. Geometrical Characteristics

To describe and analyze the saw-tooth chips, the three main parameters should be considered: the adiabatic shear band (ASB) width, the pitch of saw-tooth, and the frequency. However, due to the difficulties in measuring the ASB width, the saw-tooth degree, as well as the pitch and frequency of the saw-tooth are introduced to represent the chip’s geometrical characteristics.

**Figure 6:**
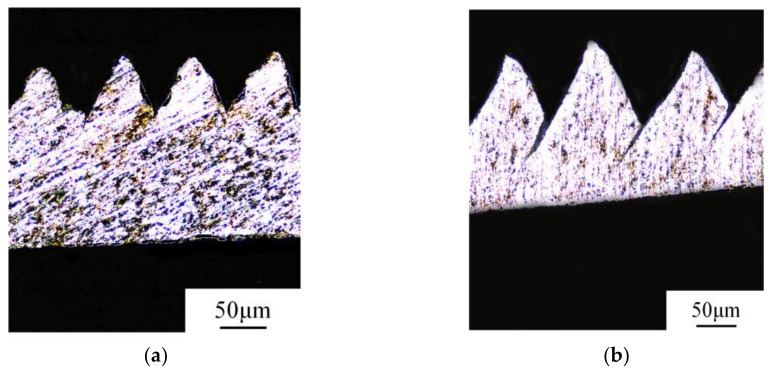

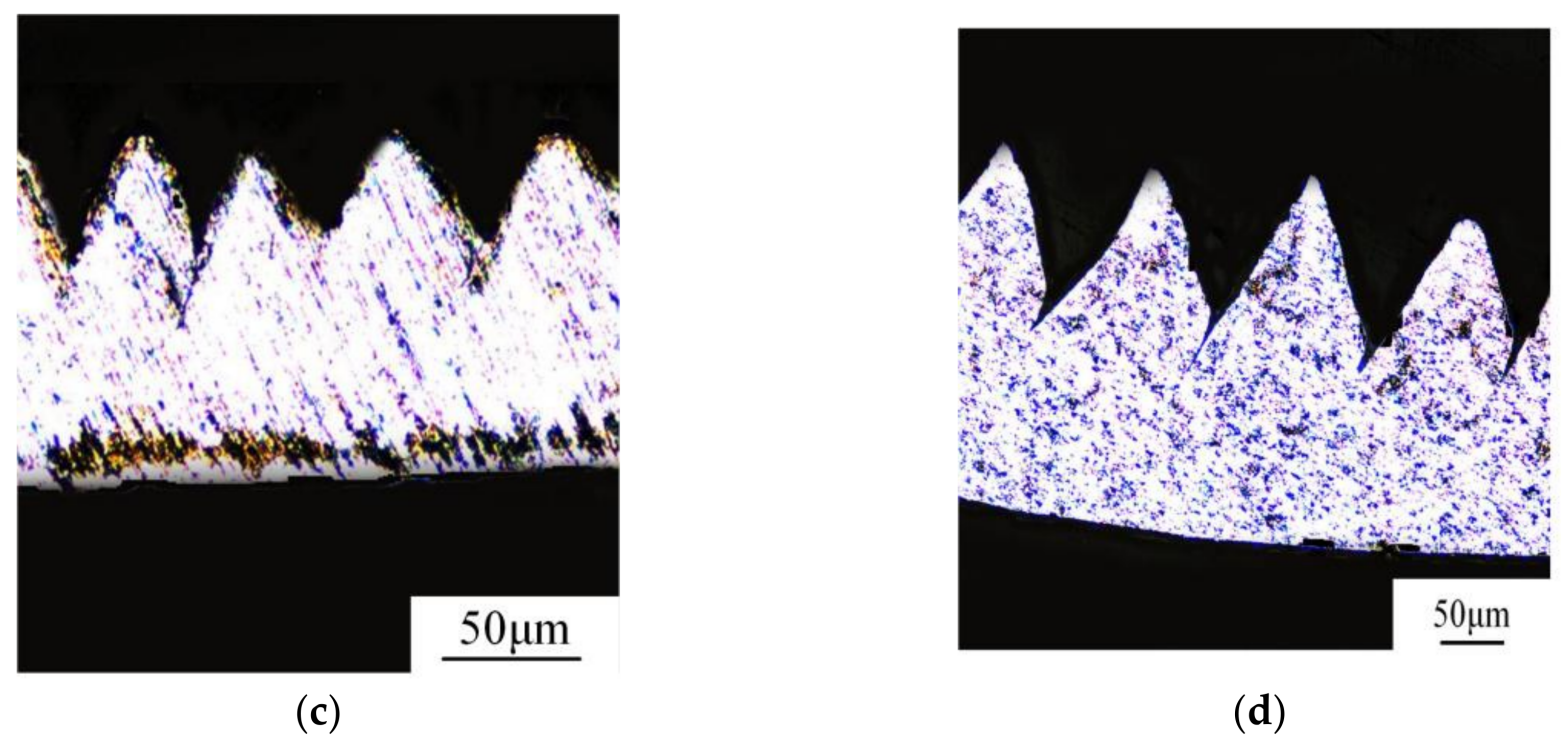
Saw-tooth chips: (**a**) *v_c_* = 50 m/min, *f_r_* = 0.2 mm/r, *a_p_* = 0.4 mm; (**b**) *v_c_* = 450 m/min, *f_r_* = 0.2 mm/r, *a_p_* = 0.4 mm; (**c**) *f_r_* = 0.1 mm/r, *v_c_* = 150 m/min, *a_p_* = 0.4 mm; (**d**) *f_r_* = 0.3 mm/r, *v_c_* = 150 m/min, *a_p_* = 0.4 mm.

The chip deformation could be directly reflected by the saw-tooth degree *G_s_*, and it was found that the saw-tooth chips were produced due to adiabatic shearing phenomena from the research of the cutting metal materials. Figure 6 presents the formed saw-tooth chips under different cutting speeds and feed rates.

From the scanning pictures of the chips, it is obvious that the saw-tooth chips could be observed under low or high cutting speed and small or large feed rate, as shown in Figure 6a–d, respectively. This is evidence that the existence of small, hard particles in the matrix material might reduce the ductility of in-situ TiB_2_/7050Al MMC, which could result in chips segmenting easily at low cutting speed.

**Figure 7:**
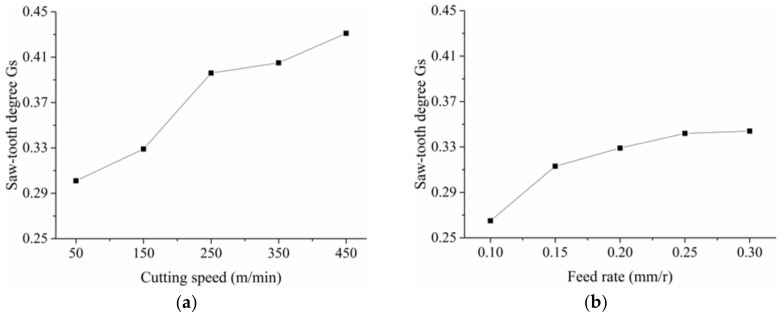
Influence of the cutting speed and the feed rate on the saw-tooth degree *G_s_*: (**a**) *f_r_* = 0.2 mm/r, *a_p_* = 0.4 mm; (**b**) *v_s_* = 150 m/min, *a_p_* = 0.4 mm.

Figure 7 shows the relationship between the saw-tooth degree *G_s_* and the cutting speed and the feed rate. With cutting speed increasing, the adiabatic shear instability takes place more easily, which resulted in larger chip deformation and increasing saw-tooth degree. As the feed rate increased, the uncut chip thickness increased accordingly. Then, the hardening during cutting was strengthened, leading to low chip flow speed. Hence, the saw-tooth degree increased as the feed rate increased.

**Figure 8:**
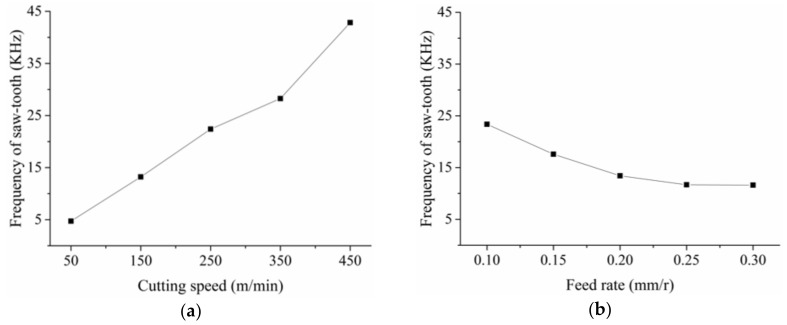
Effect of the cutting speed and the feed rate on the frequency of the saw-tooth: (**a**) *f_r_* = 0.2 mm/r, *a_p_* = 0.4 mm; (**b**) *v_s_* = 150 m/min, *a_p_* = 0.4 mm.

Figure 8 presents the change of the frequency of the saw-tooth. From Equation (2), it is obvious that the frequency *f* would increase as the cutting speed increases. However, as the feed rate increased, the frequency *f* decreased slightly, as shown in Figure 8b. With feed rate increasing, the chip deformation increased accordingly, which could also be seen from the change of saw-tooth degree in Figure 7b. Hence, the chip flow speed decreased and decelerated the saw-tooth formation process. As a result, the frequency *f* decreased as the feed rate increased. In addition, from Figure 7b and Figure 8b, it could also be found that the feed rate has a small effect on the saw-tooth degree and the frequency. On the contrary, the effect of the cutting speed on the saw-tooth degree and frequency was great.

**Figure 9:**
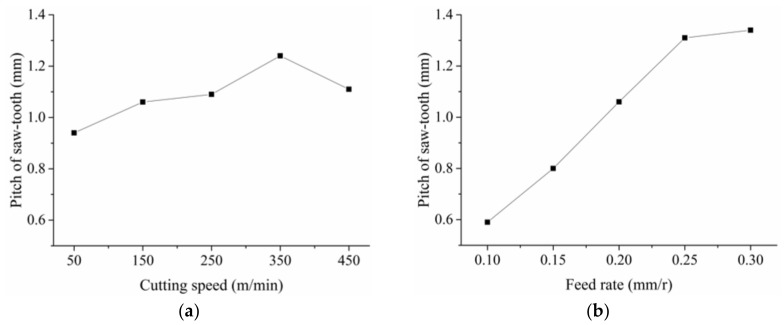
Pitch of the saw-tooth: (**a**) *f_r_* = 0.2 mm/r, *a_p_* = 0.4 mm; (**b**) *v_s_* = 150 m/min, *a_p_* = 0.4 mm.

As shown in Figure 6, the top of the saw-tooth was worn out easily during the chip metallography sample preparing process, which may result in difficulties and errors in pitch measuring. Then, the results in Figure 9 could only be used for qualitative analysis. It was clear that the effect of the feed rate on the pitch was much greater than the cutting speed. The pitch increased linearly and sharply with the increasing feed rate but only slightly with increasing speed. As a result, with the feed rate increased, the frequency discussed above decreased due to the increasing pitch of the saw-tooth. From the four pictures in Figure 6, it was obvious that the crack between the teeth increased as the speed or the feed rate increased. As the crack increased, the pitch between two tooth tops increased accordingly.

From analysis of Figure 6, Figure 7, Figure 8 and Figure 9, it is clear to see that the presence of small particles reduced the ductility of MMC, resulting in segmental chips. The saw-tooth chips were commonly observed during machining of the in-situ TiB_2_/7050Al MMC. With increasing cutting speed or feed rate, the saw-tooth degree increased accordingly. Deeper insight into the material deformation, including stress, strain rate, temperature, and shearing slip will be discussed with the simulation method in the following section.

### 4.2. Verification of Simulation Model

**Figure 10:**
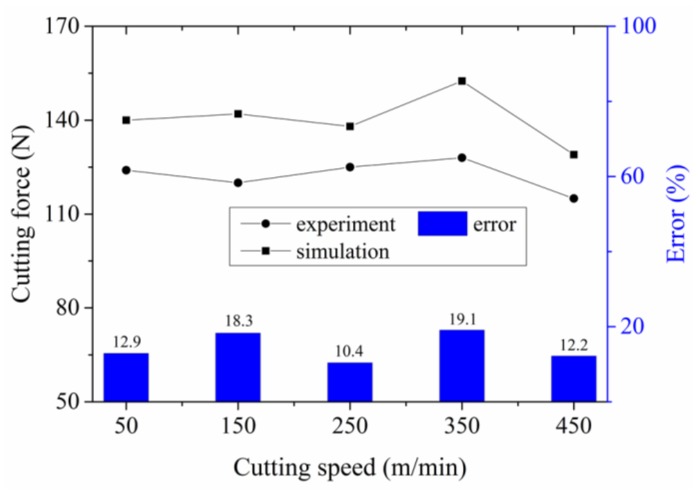
Comparison between the simulated and experimental cutting forces: *f_r_* = 0.2 mm/r, *a_p_* = 0.4 mm.

Figure 10 presents the cutting forces and relative errors of the simulation and experimental results. From our analysis, it was obvious that the simulated cutting forces were larger than that of the experiments. The J–C material constitutive equation used in the simulation was obtained from basic orthogonal cutting tests. However, the J–C damage criterion, which is more suitable for metal chip separation with using the J–C constitutive equation, is not available for this new kind of in-situ TiB_2_/7050Al MMC. Then, the shear damage, which was based on the material equivalent plastic strain, was used for chip separation. As the tool nose moved, the material in front of the tool was separated, and the material shear strain occurred. However, during simulation, the material equivalent plastic strain of the node, which was in front of the tool nose and should be damaged and separated, was usually smaller than the given value resulting in delayed fracture. This may be the reason why the simulated cutting force was larger than that of experiments. In addition, a cutting layer for anticipated separation, which did not exist in the real cutting, was applied, and this could also produce errors between the simulation and experiment results.

**Figure 11:**
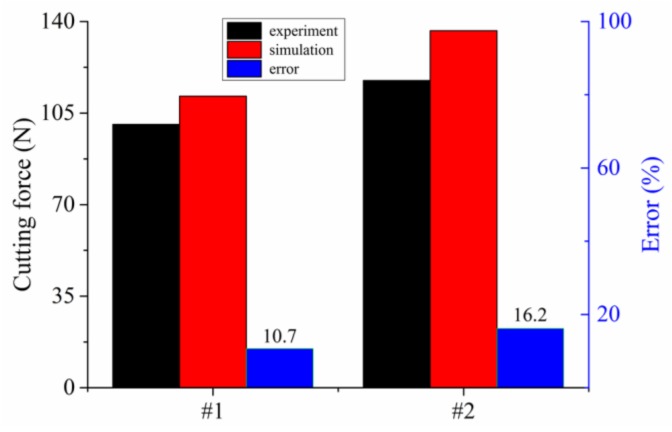
Comparison results of the verification tests between the simulated and experimental cutting forces.

Two more tests, which are shown in Table 7, were performed to verify the veracity of the obtained material constitutive equation and the FEM simulation model. Figure 11 shows the results of the verification tests between the cutting and simulation experiments. In Figure 11, the experimental cutting forces were smaller than the simulated forces due to the same reason as discussed above. It can also be seen from both Figure 10 and Figure 11 that the simulation results show good agreement with the experiment results, with relative errors being smaller than 20%.

**Figure 12:**
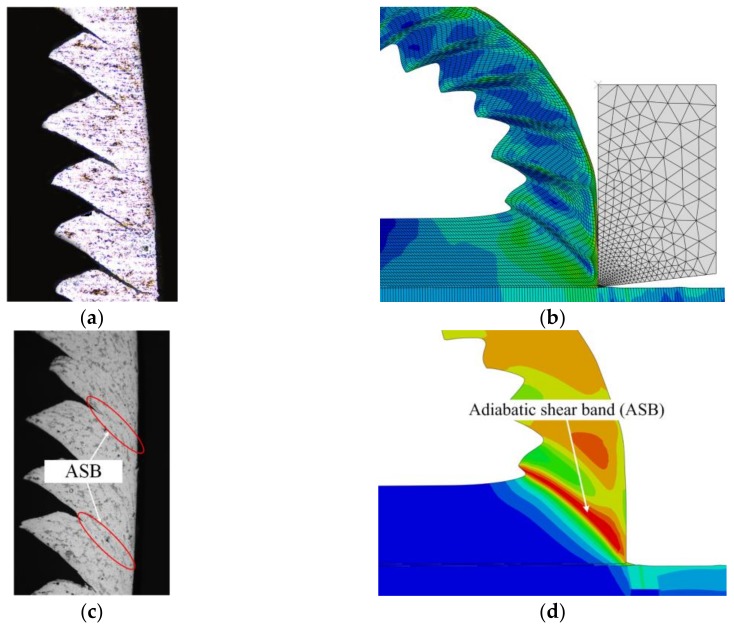
Chip morphology analysis: (**a**,**c**) experimental result; and (**b**,**d**) the finite element method (FEM) simulation result.

Meanwhile, the chip morphology of experiment and simulation was also analyzed as presented in Figure 12. From the comparison of Figure 12a,b, the shear zone was obviously found in both the chip metallographic picture and the FEM simulation result. In Figure 12c,d, the adiabatic shear band (ASB) was also found in the experimental and simulation results around the same place on the saw-tooth chips. In addition, from the cutting experiment and the simulation results, the saw-tooth chips were clearly produced, and the chip morphologies are similar. Table 8 shows the comparison of the saw-tooth geometrical characteristics, such as tooth top and root height (*h*_2_, *h*_1_), tooth vertex angle and basic angle (*θ*_2_, *θ*_1_), and pitch *d*. It can be seen that the relative errors of the saw-tooth geometrical characteristics were smaller than 25%, which indicated that the obtained material constitutive equation and the established FEM simulation model were reasonable and acceptable.

### 4.3. Chip Formation Process and Mechanisms

The research of the saw-tooth chip formation process and mechanisms is very important in the study of the chip. Due to the difficulties in measuring the plastic strain, strain rate, stress, and temperature in the chip deformation zone, the FEM and Abaqus software were applied to study the formation process and mechanisms.

**Figure 13:**
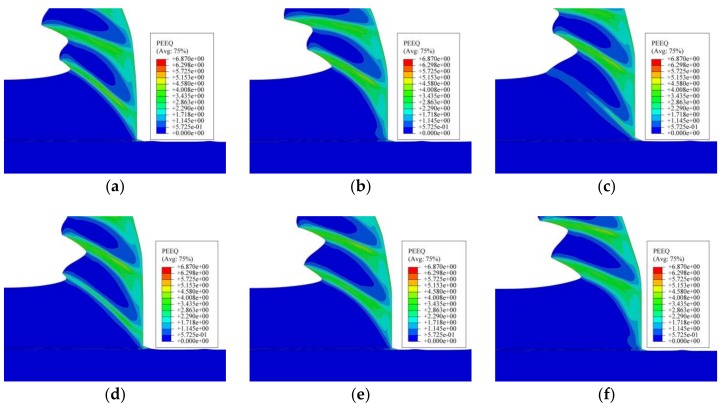
Chip formation process and the equivalent plastic strain under condition of *v_s_* = 450 m/min, *f_r_* = 0.2 mm/r: (**a**) the fully formed saw-tooth segments; (**b**) the new segment began to form; (**c**) the development of the shear plane; (**d**) adiabatic shear instability in the shear plane; (**e**) shearing slip of the chip material; and (**f**) the new saw-tooth segment was fully formed.

**Figure 14:**
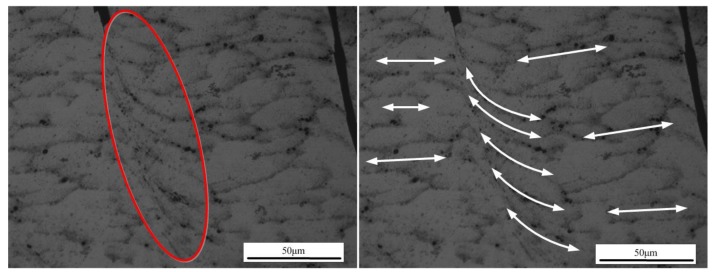
Shearing slip.

Figure 13 presents the formation process of one segment of the saw-tooth chip. In Figure 13a, one saw-tooth segment was produced initially, and there were two finished saw-tooth segments in front. With tool moving forward, the uncut chip material in front of the tool rake face was under increasing stress, as shown in Figure 13b, and the plastic deformation occurred on the material just in front of the tool nose. Meanwhile, it could be seen that the uncut chip material began to rise up and that the equivalent plastic strain of the uncut chip material in front of the tool nose raised to 1.7. Then, at the next stage, as shown in Figure 13c, the early shape of a saw-tooth segment was obvious, and the material kept rising up along the tool rake face. At the same time, the formation of the shear plane zone and the material plastic deformation could be clearly observed. In Figure 13d, the equivalent plastic strain of the shear plane was about 2.8, and the adiabatic shear instability would take place due to thermal softening and large plastic strain. Hence, in the next stage in Figure 13e, the material in the shear plane zone suffered from shearing slip, which could be obviously observed in Figure 14, and the saw-tooth segment was almost finished. In Figure 13f, the new saw-tooth segment was completed, and the next segment was to begin.

From the analysis presented in Figure 13, it could be seen that the uncut chip material in front of the tool suffered from plastic deformation. The equivalent plastic strain of adiabatic shear band was about 2.8. From Figure 13c,d and Figure 14, the formation process of the shear plane and shearing slip phenomenon was clearly observed.

**Figure 15:**
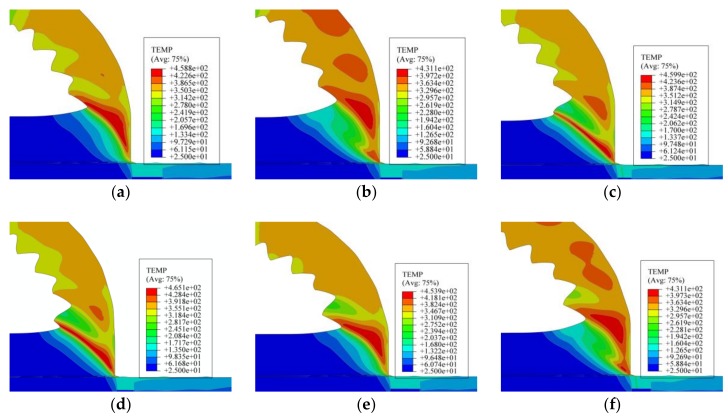
Cutting temperature under condition of *v_s_* = 450 m/min, *f_r_* = 0.2 mm/r: (**a**) The fully formed saw-tooth segments; (**b**) The new segment began to form; (**c**) The development of the shear plane; (**d**) Adiabatic shear instability in the shear plane; (**e**) Shearing slip of the chip material; and (**f**) The new saw-tooth segment was fully formed.

The cutting temperature of the chip is presented in Figure 15. Correspondingly, the six stages in Figure 15 were the same as that in Figure 13. In Figure 15a, there were three fully formed saw-tooth chip segments, and the next new segment formation was about to begin. From the temperature nephogram in Figure 15a, it can be seen that the temperature in the shear plane zone, which was about 420 °C, was higher than the other parts of the chip. With cutting tool moving, the uncut chip material was under increasing stress and was exposed to the cutting temperature, which can be seen from the increasing temperature of the uncut chip material in front of the tool nose in Figure 15b. Meanwhile, the formed chip slipped along the tool rake face, and the temperature kept increasing due to heat transmission from the shear plane. As the cutting proceeded, most of the formed chip had separated from the rake face, and the temperature decreased accordingly, as shown in Figure 15c. In this stage, the shear plane of the new segment was newly formed and the temperature in this area was much higher than in other parts. Under increasing stress, the temperature of the shear plane kept increasing, and the adiabatic shear instability occurred as presented in Figure 15d. Then, the formation of the new segment continued due to the material shearing slip, and the temperature of the shear plane rose to 450 °C. At the same time, the temperature of most of the formed chip rose to 340 °C due to heat transmission from the shear plane. In Figure 15f, the new segment was fully formed, the formed chip slipped along the tool rake face, and the next new segment was about to form.

From the temperature nephograms in Figure 15, it was obvious that the temperature in the shear plane was quite high and that the changing rule of the temperature in the shear plane was similar to the equivalent plastic strain in Figure 13. The temperature of the chip close to the tool rake face was clearly higher than that of the chip near the free surface. From the analysis of Figure 13, Figure 14 and Figure 15, it was obvious that the adiabatic shear band (ASB), adiabatic shear instability, and shearing slip phenomena existed during the saw-tooth chip formation process, which was different from cutting ex-situ SiC/Al MMC [28]. This might be due to the nanometer size and small volume fraction of particles, which resulted in similar features with cutting metals such as titanium or aluminum.

**Figure 16:**
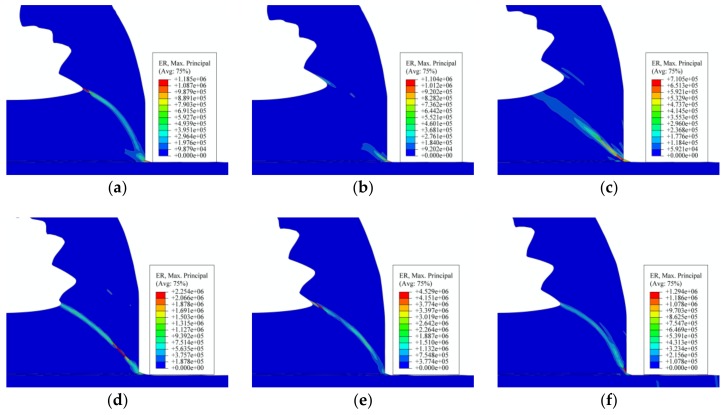
Strain rate under condition of *v_s_* = 450 m/min, *f_r_* = 0.2 mm/r: (**a**) the fully formed saw-tooth segments; (**b**) the new segment began to form; (**c**) the development of the shear plane; (**d**) adiabatic shear instability in the shear plane; (**e**) shearing slip of the chip material; and (**f**) the new saw-tooth segment was fully formed.

Figure 16 shows the strain rate during the formation process of one saw-tooth chip segment. From Figure 16b–e, it can be seen that the areas, in which the strain rate changed, were mainly in the shear plane. In addition, the strain rate increased from the tool nose to the chip free surface along the shear plane, which was the formation process of the shear plane. Accordingly, the max strain rate area marked red moved from the tool nose area to the chip free surface along the shear plane, which could be seen from Figure 16b–e. Figure 16a,f, which represented the completion stage of the formed segment, showed that the strain rate value of the shear plane did not decrease sharply. From Figure 16a–d, it could be concluded that the strain rate of the first segment did not decrease to zero until the adiabatic shear instability occurred in the next segment, as shown in Figure 16d. This indicated that the changing of the strain rate was kind of later than that of the equivalent plastic strain and temperature in the FEM simulation results. In addition, the movement of the max strain rate area along the shear plane was also evidence of the shearing slip. Moreover, as presented in Figure 16, the max strain rate in the shear zone during the cutting process was 4.5e^+6^. It was much higher than that in Split Hopkinson Pressure Bar (SHPH) test, which was one of the reasons why the material constitutive model was obtained from the cutting tests.

**Figure 17:**
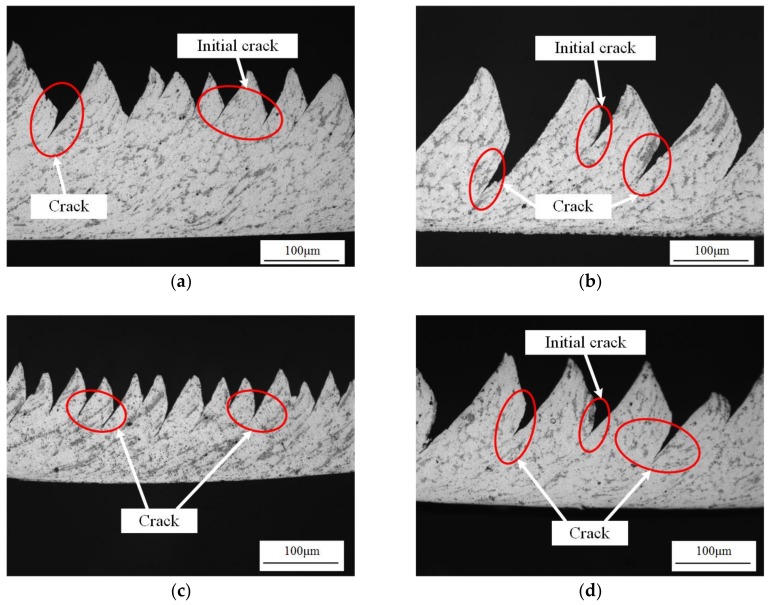
Cracks in the formed chips: (**a**) *v_s_* = 50 m/min, *f_r_* = 0.2 mm/r; (**b**) *v_s_* = 450 m/min, *f_r_* = 0.2 mm/r; (**c**) *f_r_* = 0.1 mm/r, *v_s_* = 150 m/min; (**d**) *f_r_* = 0.3 mm/r, *v_s_* = 150 m/min.

However, there were some differences between the simulation and experimental results, such as cracks due to the application of a cutting layer along the path of anticipated separation, and the shear damage instead of J–C damage criterion. In the simulation results in Figure 13, Figure 15, and Figure 16, there are no obvious cracks between the formed chip segments. Nevertheless, in the experimental results in Figure 17, obvious cracks between the chip segments were clearly observed under either high cutting speed or low speed and large feed rate or small feed rate.

In Figure 17, the cracks extended along the shear plane from the tooth root to the joint inner surface. With the cutting speed or feed rate increasing, the crack length increased, resulting in increasing saw-tooth degrees, as presented in Figure 7. Due to the existence of the small, hard particles, the ductility of in-situ TiB_2_/7050Al MMC was reduced. The initial crack would produce when the shear plane was to form as shown in Figure 13d and Figure 15d. Then, under great stress and high temperature, the crack extended with the formation of the shear plane. As a result, the segment was fully formed under the combined effect of the adiabatic shear instability, shearing slip, and crack extension.

## 5. Conclusions

From the cutting experimental and FEM simulation results of chips of in-situ TiB_2_/7050Al MMC with PCD tools, the following conclusions can be drawn:

(1) With data from basic orthogonal cutting experiments and the genetic algorithm, the Johnson–Cook material constitutive equation of in-situ TiB_2_/7050Al MMC could be determined. In the verification of the experimental and simulation results, the relative error of the cutting force is less than 20%, and the chip morphology is very similar, which indicates the material constitutive constants (*A*, *B*, *C*, *m*, and *n*) are of high reliability.
(2) From the analysis of the chips, it was found that the saw-tooth chips were commonly found under either low cutting speed (50 m/min) or high speed (450 m/min) and small feed rate (0.1 mm/r) or large feed rate (0.3 mm/r), which was different from cutting the non-reinforced aluminum alloy. It was found that the existence of small hard particles reduced the ductility of the MMCs and resulted in segmental chips.
(3) The relationship between the cutting parameters and chip geometrical characteristics were analyzed. With cutting speed increasing, the saw-tooth degree and the frequency of saw-tooth increased quickly due to the adiabatic shear instability and larger chip deformation. However, it was found that the feed rate did not make much difference to the saw-tooth degree and the frequency due to the low chip flow speed and the decelerated saw-tooth formation process. On the contrary, the influence of the feed rate on the pitch was much greater than that of the cutting speed. As the feed rate increased, the pitch increased linearly and sharply. In addition, it was found that the crack between teeth increased as the cutting speed or feed rate increased.
(4) The formation process and mechanisms of one saw-tooth chip segment was analyzed. It was found that the chip formation mechanisms of in-situ TiB_2_/7050Al MMC included plastic deformation, adiabatic shear, shearing slip, and crack extension. From the analysis of the equivalent plastic strain, temperature, and strain rate, the formation process of the shear plane was obviously observed.
(5) The change of the cutting temperature, strain rate, and equivalent plastic strain was mainly in the shear plane zone, which is evidence of the adiabatic shear band, plastic deformation, and material shearing slip. The adiabatic shear band was observed and had much to do with the chip formation mechanisms, which were different from that in cutting ex-situ SiC/Al MMCs.
(6) By comparison of the equivalent plastic strain, temperature, and strain rate under the same formation stage, it was found that, in the FEM simulation results, the change of the equivalent plastic strain and temperature was simultaneous. However, the changing of strain rate is kind of later than that of the equivalent plastic strain and temperature.

Continuing works will focus on the improvement of the FEM simulation model, especially on determining proper material failure criteria. Due to the huge size differences between particle size and uncut chip thickness, the particles were not presented in our FE model. Therefore, research including the impact of nanometer particles and particle-matrix interfaces are necessary in future studies with improved simulation models or molecular dynamic simulation techniques.

## Figures and Tables

**Table 1:** Specifications of the polycrystalline diamond (PCD) tool.

Specifications	Rake Angle	Flank Angle	Cutting Edge Radius
PCD tool	0°	10°	2 μm

**Table 2:** Material physical properties of PCD tool.

Density	Poisson’s Ratio	Elastic Modulus	Specific Heat	Thermal Conductivity	Thermal Expansion
3.52 g/cm^3^	0.1	1050 GPa	420 J/(kg·K)	1000 W/(m·K)	2.0 × 10^−6^/K

**Table 3:** Chemical composition of the matrix material.

Element	Zn	Zr	Mg	Cu	Al
Weight (%)	6.2	0.12	2.2	2.3	Balanced

**Table 4:** Physical properties of in-situ TiB_2_/7050Al metal matrix composite (MMC).

Density	Poisson’s Ratio	Elastic Modulus	Shear Modulus	Yield Strength	Melting Point
2.90 g/cm^3^	0.33	78 GPa	29 GPa	639 MPa	476 °C

**Table 5:** Cutting parameters.

Factor	Notation	Level				
		1	2	3	4	5
Cutting speed (m/min)	*v_s_*	50	150	250	350	450
Feed rate (mm/r)	*f_r_*	0.1	0.15	0.2	0.25	0.3
Cutting depth (mm)	*a_p_*	0.2	0.4	0.6	0.8	1.0

**Table 6:** Obtained material constants of in-situ TiB_2_/7050Al MMC.

*A* (MPa)	*B* (MPa)	*C*	*m*	*n*
630	1127	0.004	2.4	0.972

**Table 7:** Verification tests.

No.	*v_s_* (m/min)	*f_r_* (mm/r)	*a_p_* (mm)
#1	400	0.22	0.4
#2	300	0.28	0.4

**Table 8:** Relative errors of chip geometrical characteristics between the experiment and simulation.

No.	Result	*h*_1_	*h*_2_	*θ*_1_	*θ*_2_	*d*
#1	Experiment	0.136 mm	0.243 mm	52.325°	62.165°	0.111 mm
	Simulation	0.165 mm	0.273 mm	60.189°	76.170°	0.136 mm
	Relative error (%)	21.3	12.3	15.0	22.5	22.5
#2	Experiment	0.178 mm	0.266 mm	53.582°	64.463°	0.124 mm
	Simulation	0.207 mm	0.259 mm	59.289°	80.523°	0.140 mm
	Relative error (%)	16.3	2.6	10.7	24.9	12.9
